# Ranking’s Half-Yearly Abstract

**Published:** 1860-05

**Authors:** 


*Ranking's Half-Yearly Abstract of the Medical Sciences*, Part the Thirtieth, is also before us, through the kindness of the American publishers, Lindsay & Blakiston, Philadelphia.

Our readers will bear in mind, that for *four dollars*, sent to the office of the MONTHLY, either of the above reprints, with the MONTHLY, will be sent one year; making decidedly the cheapest, and yet the best, medical reading to be had in the country—*seventeen hundred* pages for four dollars. O. C. G.

